# Anatomical Changes in the Peel of Sun-Damaged Pomegranates (*Punica granatum* L. cv. Hicaznar)

**DOI:** 10.3390/plants15060987

**Published:** 2026-03-23

**Authors:** Keziban Yazıcı, Muhammad Tanveer Altaf, Lami Kaynak

**Affiliations:** 1 Department of Horticulture, Faculty of Agriculture and Natural Sciences, Recep Tayyip Erdoğan University, Rize 53300, Türkiye; 2 Department of Field Crops, Faculty of Agriculture, Recep Tayyip Erdoğan University, Rize 53300, Türkiye; muhammadtanveer.altaf@erdogan.edu.tr; 3 Department of Horticulture, Faculty of Agriculture, Akdeniz University, Antalya 07070, Türkiye

**Keywords:** pomegranate, sunburn, peel, anatomy, cuticle, epidermis, high temperature

## Abstract

Pomegranate (*Punica granatum* L.) is a major fruit crop in tropical and subtropical regions, but changing climatic conditions—especially rising temperatures and intense solar radiation—are increasing physiological disorders. Sunburn, a key heat- and light-induced disorder, causes peel discoloration and tissue damage. This results in significant yield loss and reduced fruit quality. The objective of this study was to characterize sunburn-induced anatomical changes in the widely grown, highly sensitive *Hicaznar* cultivar in Türkiye, and to identify the optimal phenological stage for the application of sunburn-preventive practices. For this purpose, pomegranate fruit peels were fixed in FAA (Formalin–Acetic Acid–Alcohol) solution, embedded in paraffin blocks, and sectioned at a thickness of 5–7 µm. The sections were stained using the hematoxylin–eosin method and examined under a light microscope. The images captured with a digital camera wereanalyzed and revealed that sunburn damage in the pomegranate peel first appears in the cuticle layer, followed by disruption and fragmentation of the cutaneous and epidermal layers beneath it, and ultimately leads to damage of the parenchyma cells. Furthermore, Light microscopy showed that before visible discoloration, cells near the epidermis undergo phenolic accumulation, cell-wall thickening, and lignification, which are early indicators of sunburn. These microscopic changes provide early diagnostic features for detecting sunburn damage before external symptoms manifest. The study concluded that anatomical changes begin before the visible symptoms of sunburn appear on the fruit, and the most appropriate timing for applying preventive measures against sunburn has been identified. Light microscopy showed that before visible discoloration, cells near the epidermis undergo phenolic accumulation, cell-wall thickening, and lignification, which are early indicators of sunburn.

## 1. Introduction

Pomegranate (*Punica granatum* L.) is native to the regions of Persia and Central Asia [1], from where it spread to the Mediterranean, Middle East, and parts of Asia [2,3,4]. Although several centers of origin have been proposed, most studies agree that the species has deep genetic and cultural roots across these regions [3,5,6,7]. The genus *Punica* includes only two species: the cultivated *P. granatum* and *P. protopunica*, which naturally occurs on the Socotra Islands of Yemen and is considered its closest wild relative. Understanding the crop’s broad adaptation and long domestication history is important, as these factors influence its sensitivity to environmental stresses such as sunburn [8,9].

The domestication of the pomegranate began in the Neolithic era [3,10], initially in the Transcaucasia–Caspian region and northern Türkiye [11,12]. Despite long-term cultivation and selection, there is minimal morphological difference between wild and domesticated forms, with major changes being in fruit size, seed hardness, and color [2,10,12].

In recent years, pomegranate has gained widespread global recognition, largely due to its rich composition of bioactive compounds, such as polyphenols, flavonoids, and tannins [13]. These compounds exhibit str ong antioxidative and anti-inflammatory properties and are associated with reduced risks of chronic diseases, including cardiovascular disorders and certain cancers [14,15]. This growing popularity reflects not only its functional health benefits but also its cultural and economic significance in both traditional and modern contexts.

Global pomegranate market size was worth around $208.9 million in 2019 and Türkiye led global pomegranate exports with 155,000 tons, accounting for 23% of worldwide exports (626,336 tons) [16]. World pomegranate production was about 8 million tons in 2019 [16,17,18]. The main pomegranate-producing countries were India and China, which recorded a production exceeding 3 and 1 million tons, respectively [16,19]. Meanwhile, pomegranate production in Türkiye reached 638,821 tons in 2023, according to the Turkish Statistical Institute [20].

In addition to its nutritional and cultural significance, pomegranate cultivation is particularly vulnerable to the increasing challenges posed by global climate change. Rising temperatures, irregular rainfall patterns, and the intensification of solar radiation are expected to increase the prevalence of sunburn across major pomegranate-growing regions [21]. This risk is particularly significant in Mediterranean and arid climates, where prolonged heat waves have already caused substantial yield and quality losses in other fruit crops, including apples, grapes, and peaches [22]. Addressing these challenges requires not only improved orchard management practices but also a deeper understanding of the anatomical and physiological mechanisms underlying fruit susceptibility to environmental stressors [23]. In this regard, anatomical studies provide a crucial scientific foundation for linking structural changes in the peel to broader physiological processes, thereby enabling the development of more effective strategies for early detection and mitigation [24].

Despite its increasing production and consumption, sunburn represents a major physiological disorder in pomegranate cultivation, especially in arid and semi-arid climates [25]. This damage results from intense environmental stressors high temperatures, ultraviolet (UV) radiation, and solar radiation leading to visual and anatomical damage on fruit surfaces. Sunburn can cause the peel color to range from brown to black and is often accompanied by internal dehydration and reduced grain quality [26,27]. Sunburn in pomegranates is a physiological disorder caused by excessive heat and light, leading to oxidative damage, increased membrane damage, and reduced fruit quality [28]. Antioxidants like phenols and flavonoids increase in response, but severe sunburn decreases their overall content. Crop losses due to sunburn have been reported to reach 40–50%, particularly in Mediterranean regions like Türkiye [29].

To mitigate sunburn damage, techniques such as evaporative cooling, shade netting, kaolin-based reflective sprays, and antioxidant treatments (e.g., Vitamin E, Vapogard) have been applied with varying success. However, preventive strategies must be employed before visible symptoms occur, as cellular and anatomical damage often precedes external signs [30]. Sunburn-induced damage is strongly linked to oxidative stress and photoinhibition, which are exacerbated by high light intensity and prolonged exposure [31]. In fruit crops, sunburn is known to develop through a combined effect of excessive visible and UV radiation together with elevated fruit temperature, a synergistic interaction that triggers photodamage, membrane destabilization, and oxidative stress [32]. The generation of reactive oxygen species (ROS) such as hydrogen peroxide and superoxide radicals damages cellular membranes, pigments, and nucleic acids [33,34]. Plants defend against such damage through antioxidant systems composed of enzymatic and non-enzymatic molecules, including ascorbic acid, glutathione, tocopherols, and flavonoids.

**Figure 1:**
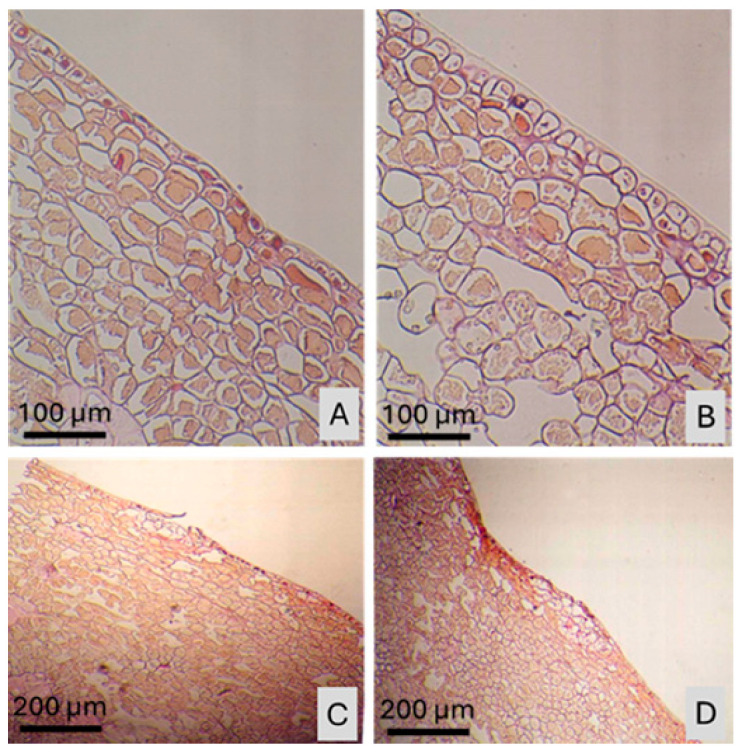
Light micrographs of normal pomegranate (*Punica granatum* L. cv. Hicaznar) peel tissues not exposed to sun damage. (**A**,**B**) Transverse sections showing intact epidermis and flavedo layers at ×20 magnification. (**C**,**D**) General anatomical organization of peel tissues at ×10 magnification. Scale bars = 100 µm (**A**,**B**) and 200 µm (**C**,**D**).

Anatomical adaptations, such as cell wall thickening, cuticle modifications, and lignification, play a critical role in plant resilience to environmental stress [35,36]. The peel of pomegranate fruit consists of a highly durable and thick cuticle layer, providing resistance against many external factors (Figure 1). However, when exposed to high temperatures and direct sunlight, sunburn may occur on the fruit peel. Despite numerous studies on pomegranate physiology, few have examined the anatomical structure of the peel under sunburn stress. Understanding the changes in cell structure and tissue morphology can offer insights into early detection and more effective mitigation strategies. Similar anatomical responses to sunburn have been reported in other fruit species. For example, in apple (*Malus domestica*), sun-exposed skin shows ultrastructural damage such as cuticle thinning and epidermal collapse [37]. In peach (*Prunus persica*), histological studies have revealed cell wall degradation and epidermal disintegration under heat stress [38]. Grapes (*Vitis vinifera*) exhibit increased flavonol degradation and epidermal damage in response to sunlight exposure [39], while in kiwifruit (*Actinidia deliciosa*), lignification and phenolic accumulation were observed in peel tissues subjected to high solar radiation [40]. These findings support the relevance of anatomical studies across fruit crops to better understand and prevent sunburn-related quality losses. In this study, we investigate the microscopic anatomical differences between sun-damaged and undamaged peels of ‘*Hicaznar*’ pomegranates using light microscopy. In this study, it was aimed to relate anatomical findings to practical orchard management by determining when sunburn injury first occurs, thereby providing growers with the most appropriate time window for implementing protective measures.

## 2. Results and Discussion

In pomegranates, sunburn leads to a sequence of color changes on the fruit skin, including yellowing, browning, and darkening. Light microscope images have shown that before any color change occurs on the fruit skin, sunburn manifests as a buildup of phenolic substances, thickening of cell walls, and lignification of cells, particularly in those near the epidermis (Figure 1). Protective practices against sunburn in pomegranate should be applied before this stage, while the fruit peel is still green and the color change (to red or pink) has not yet begun.

**Figure 2:**
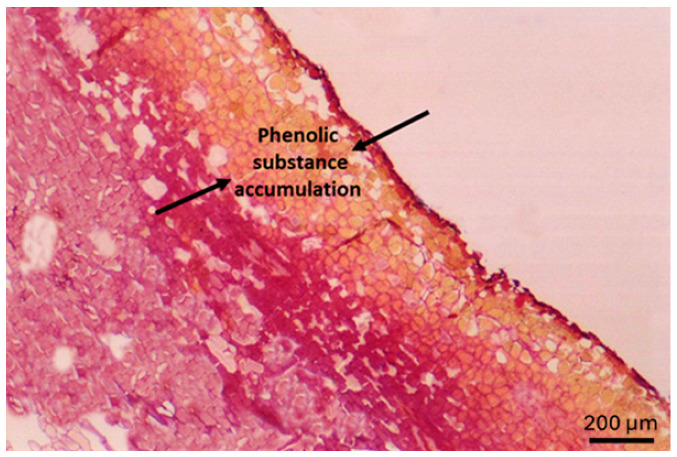
Light micrograph of pomegranate (*Punica granatum* L. cv. Hicaznar) peel tissue showing early accumulation of phenolic compounds in epidermal and sub-epidermal cell layers under sun exposure. Phenolic-rich regions are indicated by arrows. At this stage, no externally visible sunburn symptoms are detectable on the fruit surface. Scale bar = 200 µm.

In *Punica granatum* L. cv. Hicaznar, severe sunburn causes progressive discoloration of the fruit peel from yellowing to browning and eventually dark necrotic patches. Anatomical examination revealed that this damage disrupts the outermost peel layers: the cuticle and epidermis develop cracks, and the superficial cells show signs of degradation, while deeper peel tissues remain largely intact, indicating that cell collapse is confined to the most external strata. These structural changes coincide with increased peel hardness, likely due to dehydration and tissue desiccation induced by intense solar exposure, and the fragmentation of the cuticle and widening of microcracks are more pronounced in the equatorial and calyx regions, which feature thinner peel and denser lenticels (Figure 2). Similar findings have also been reported by other researchers [41].

**Figure 3:**
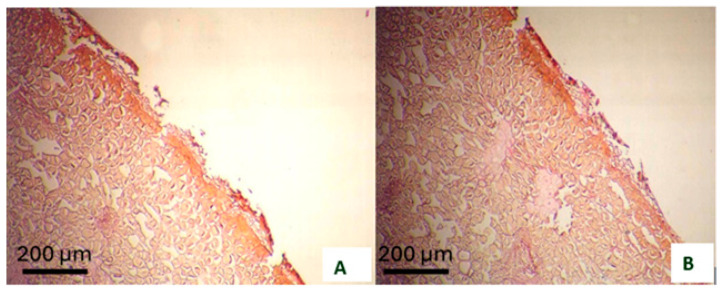
Light micrographs of sunburn-affected pomegranate (*Punica granatum* L. cv. Hicaznar) peel surface. The cuticle shows early degradation characterized by micro-cracking and partial detachment from the underlying epidermis (**A**,**B**). Epidermal cells beneath the damaged cuticle exhibit initial signs of cell wall collapse and deformation. Scale bar = 200 µm.

The homogeneity, thickness, and composition of the cuticle layer, the presence of trichomes on the fruit surface, and the quantity of surface pigments contribute to protection against sunburn by reducing fruit temperature through increased reflection of visible and infrared light. The cuticle layer acts as a protective barrier that reduces compounds harmful to cells before they cause damage. In pomegranates, sunburn initially occurs in the cuticle layer. As a result, the cuticle layer begins to degrade. Furthermore, the underlying epidermal layer also starts to disintegrate and break apart (Figure 3).

These findings are consistent with the studies by Polito et al. [36], who also observed that phenolic substances accumulate in plant cells subjected to stress conditions. Excessive sunlight leads to the formation of active oxygen species (hydrogen peroxide, superoxide, etc.), which, through a series of reactions, cause cellular damage and lead to photo-oxidative damage. These compounds are highly reactive molecules with one or more unpaired electrons that cause oxidative damage to cellular membranes and DNA. Rather than altering the genetic code itself, they oxidize DNA bases and can induce single- or double-strand breaks. In Fuji apples, this condition manifests as a color change from yellow to brown, while in Granny Smith apples, the first indication of photo-oxidative damage is a whitening of the fruit surface [42].

**Figure 4:**
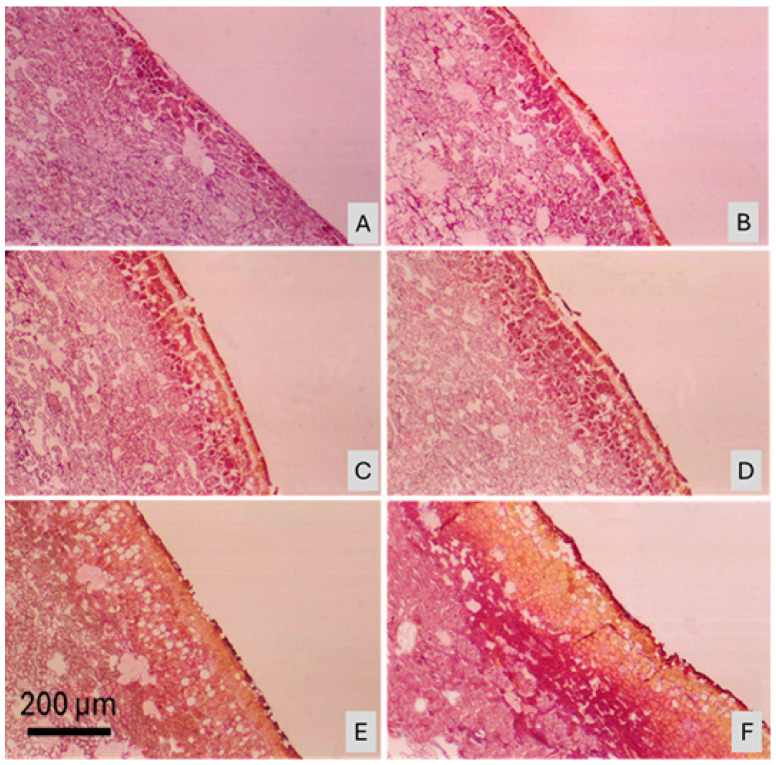
Light micrographs illustrating anatomical differences between healthy pomegranate (*Punica granatum* L. cv. Hicaznar) peel and peel tissues affected by progressive stages of sunburn. A continuous cuticle layer and a single, orderly row of epidermal cells are observed in healthy peel tissue (**A**). Stages (**B**–**D**) show cuticle disruption, epidermal cell fragmentation, cell wall thickening, phenolic compound accumulation, and increased lignification. Stages (**E**,**F**) represent the phase immediately preceding externally visible discoloration. Scale bar = 200 µm.

As a result of our anatomical studies on the fruit peel that was directly exposed to sunlight, especially under high temperature and solar radiation, it was determined that the regions of the peel where phenolic compounds accumulated were stained in a yellowish-brown hue using Harris’s hematoxylin and eosin staining method. Based on the conducted research, it was found that the outermost layer (pericarp) of a healthy pomegranate fruit peel consists of a cuticle layer, with a single layer of epidermal cells arranged directly underneath (Figure 4A). In contrast, in the fruit peel affected by sunburn at the same developmental stage, the epidermal cells are fragmented, dark spots appear, cell walls of the epidermal cells become thickened, and phenolic compound accumulation is observed (Figure 4B–F). These structural changes are also stained using Harris’s hematoxylin and eosin method. Such features were not observed in healthy fruits.

The anatomical alterations identified in the present study, including cuticle degradation, epidermal collapse, and localized phenolic accumulation, are consistent with previously defined temperature-dependent sunburn stages in pomegranate. Previous quantitative work has shown that sunburn incidence increases sharply when fruit surface temperature (FST) exceeds 41–47 °C, and that FST is strongly correlated with solar radiation and air temperature [43]. These thresholds indicate that the microscopic symptoms observed here correspond to the physiological transition from browning to necrotic and severe sunburn injury.

In a study examining the anatomical, biochemical, and physiological changes occurring on the fruit surface of the sunburned Fuji apple variety, it was reported that the most prominent symptom of sunburn caused by intense sunlight was the lightening of fruit surface color. For instance, in sunburned fruits, the red coloration decreased while yellow coloration increased. It was noted that chlorophyll and other pigments found in leaves and fruits influence photooxidative damage through their roles in light absorption and reactive oxygen species (ROS) dynamics [44]. Chlorophyll normally converts solar energy into chemical energy. However, under excessive sunlight conditions, chlorophyll can become harmful by exciting its own electrons, which are then transferred to reactive oxygen species (ROS) dangerous oxidants. Anthocyanins were found to eliminate these ROS by absorbing excessive UV radiation, and an increase in some flavonoid compounds on the fruit surface was observed as a stress response to UV radiation, providing protection against sunburn. It was also determined that in fruits exposed to sunlight, the amount of β-carotene, a carotenoid was higher compared to fruits grown in shade. These findings suggest that the color change observed in sunburned fruits is a kind of protective reaction [44].

**Figure 5:**
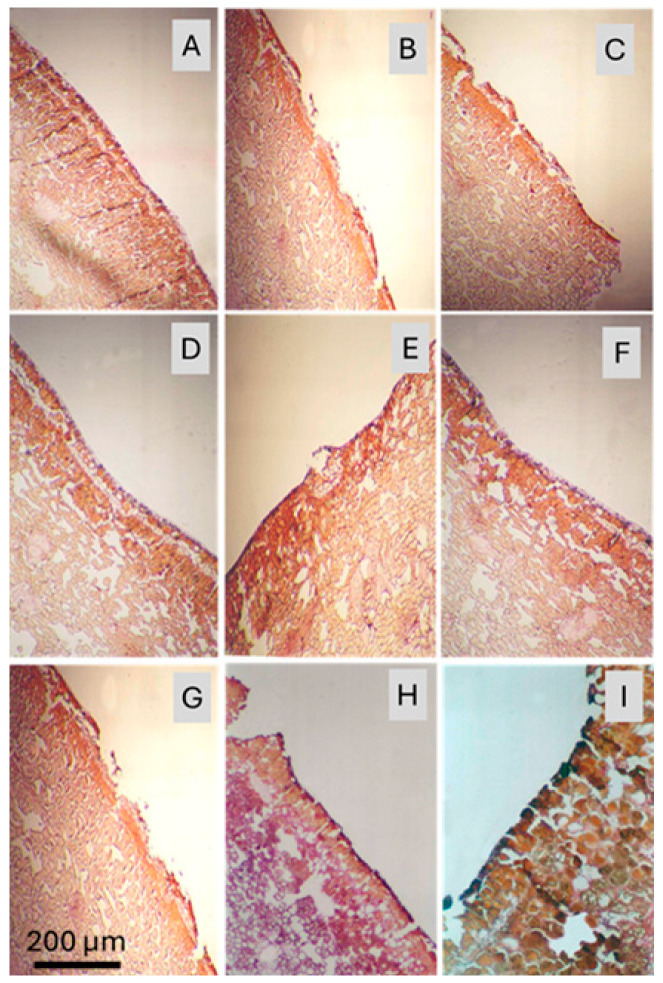
Progressive stages of sunburn-induced tissue degradation in pomegranate (*Punica granatum* L. cv. Hicaznar) peel visualized using the cellulose acetate method. In stages (**A**–**C**), the cuticle layer is disintegrated and the epidermal layer begins to break down. In stages (**D**–**F**), tissue degradation extends into the parenchyma cells. In stages (**G**–**I**) complete cellular disintegration and enlargement of intercellular spaces were observed. Scale bar = 200 µm.

In the early stages of sunburn, the epidermal cells on the fruit peel begin to show signs of disintegration and fragmentation (Figure 5A–E). In the more advanced stages of sunburn, a large area of the peel surface retains only the epidermis, while the cuticle layer has completely disappeared (Figure 5F–I). On the opposite side of the same fruit, however, the cuticle layer remains intact, with only thinning and partial disintegration observed in the areas where spotting has begun. In addition, in the sunburned fruit peel, sclerenchyma cells appear larger and more numerous. The color changes observed in the cells of sunburned fruit peel also serve as indicators of damage to pigmentation. This study confirms that cellular organelles are damaged before any visible symptoms of sunburn appear. These findings highlight the importance of implementing preventive measures prior to the development of visible sunburn symptoms on the fruit. When we look at the microscopic studies, it has been observed that in pomegranate peel affected by sunburn, the parenchyma cells shrink, and the intercellular spaces increase. In the later stages of sunburn, it was found that the lenticels, sclerenchyma cells, and vascular bundles between the parenchyma cells were broken down (Figure 5H,I).

Importantly, the early anatomical indicators identified in this study—such as phenolic accumulation, cell-wall thickening, and initial cuticle fragmentation—highlight that sunburn stress begins well before visible discoloration appears on the fruit surface. These findings have practical significance for orchard management. Detecting these changes at an early stage suggests that preventive measures such as shading, evaporative cooling, reflective kaolin sprays, or antioxidant applications must be initiated before external symptoms emerge. Therefore, the anatomical markers described here can serve as an early-warning framework, enabling growers to time their interventions more accurately and reduce yield and quality losses.

In the stage where the fruit peel turns completely black, it was observed that the cells forming the peel completely lose their function, the damage spreads through the peel and reaches the seeds, where a dry, colorless, hard formation occurs. When a fruit affected by sunburn was cut in half, it was seen that the seeds on the sunburned side were colorless and dried out, while the seeds on the intact side were not affected in this way.

There have been very few anatomical studies reported on the effects of sunburn on fruit, and particularly on the peel anatomy. However, the findings of studies conducted on various fruit species affected by sunburn, such as those by Barber and Sharpe [45], Andrews and Johnson [44,46], Renquist et al. [47], and Polito et al. [36], were found to be similar to our findings.

The anatomical injury patterns observed in the present study correspond closely to previously defined environmental thresholds, where browning begins at FST ≈ 35 °C, necrotic browning at 40 °C, and severe blackening at ≥45 °C [48]. This alignment confirms that cuticular disruption and epidermal collapse are thermally driven processes tightly linked to FST load.

Studies have reported that in fruits affected by sunburn, there is collapse in the epidermal cells, and changes in the cytoplasmic contents of the cells. In mature fruits where the burn has progressed, it has been noted that the epidermal cells completely collapse and disintegrate, the walls of subepidermal cells thicken, parenchyma cells elongate, the number of cells increases, and the average cell diameter grows. On the intercellular surface, an increase in pectic substances, accumulation of fibrous cellulose materials, and suberization in the cell wall have been detected. It was also found that the cuticle layer thinned and completely disappeared in the part of the peel with more severe sunburn [30,37,38,40].

In a study examining the anatomy of sunburn-affected strawberries, it was reported that in the fleshy receptacle tissue (pseudofruit), epidermal cells condense and form a dark mass, the cuticle layer is damaged and disintegrates, and phenolic substances accumulate in the subepidermal cells [31,36]. The epidermal cells of the bronzed receptacle tissue showed collapse in the radial plane compared to the healthy tissue [36]. Andrews and Johnson [30] suggested that the anatomical changes in the fruit peel resulting from sunburn represent a type of response by the plant. They stated that the waxy cuticle layer serves as the first defense against intense sunlight and high temperatures by reducing solar radiation damage before it reaches the cells. The accumulation of phenolic substances in the cells beneath the cuticle layer is also considered an indication of an antioxidant protection mechanism. Similarly, Renquist et al. [46] reported that in red raspberries, high temperatures and UV radiation caused color loss in the cuticle layer, with the color change appearing as yellowing and browning.

**Figure 6:**
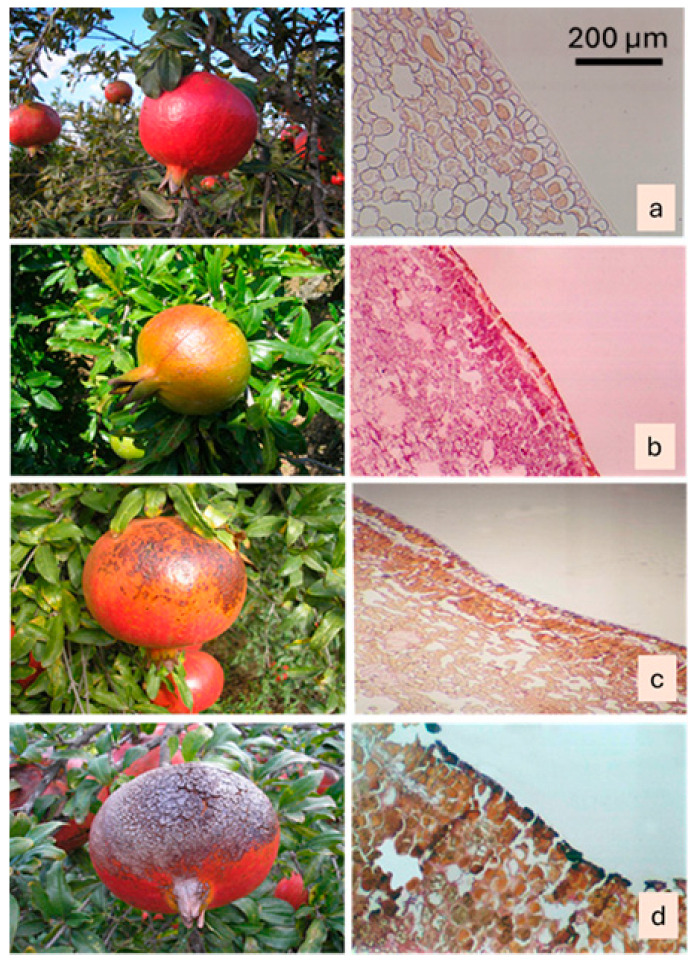
Macroscopic and microscopic comparison of pomegranate (*Punica granatum* L. cv. Hicaznar) peel tissues across increasing degrees of sunburn severity. Intact fruits and corresponding peel anatomy show a continuous cuticle and well-organized epidermal cells (**a**). Early sunburn is associated with initial surface discoloration and localized cuticle thinning (**b**). Moderate sunburn is characterized by the appearance of dark brown spots, cuticle disruption, and epidermal cell deformation (**c**). Severe sunburn exhibits complete surface darkening, fragmentation of the outer peel layers, and extensive cellular collapse (**d**). Scale bar = 200 µm.

Taking into account the color changes and the anatomical studies conducted during these stages in pomegranates, sunburn damage can be categorized as: 1. undamaged (Figure 6a), 2. slight burn, 3. moderate burn, and 4. severe burn. In fruits with slight burns, sunburn has just started and appeared as yellowing (Figure 6b); in moderately burned fruits, dark brown and black spots began to form along with the yellowing (Figure 6c); and in severely burned fruits, the peel surface has completely turned black and taken on a fragmented appearance (Figure 6d). Kaolin treatments applied at stages of moderate and severe sunburn in pomegranates were found to be ineffective in preventing sunburn, while treatments applied to fruits with slight burns were somewhat effective [47]. Therefore, both the treatments and the findings from anatomical studies suggest that applications to prevent sunburn should be initiated during the period when burn symptoms are not yet visible.

## 3. Materials and Methods

### 3.1. Plant Material

**Figure 7:**
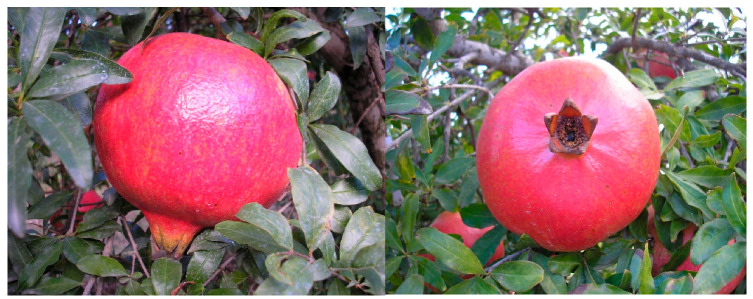
Fruits of the Hicaznar pomegranate cultivar used in the experiment.

The plant material consisted of fruits from 14-year-old pomegranate trees of the cultivar *Hicaznar* grown at the experimental orchards of the Batı Akdeniz Agricultural Research Institute (BATEM), Serik-Kayaburnu, Antalya, Türkiye. The *Hicaznar* cultivar belongs to the family Punicaceae, genus *Punica*, which includes *Punica granatum* L., the main cultivated species worldwide (Figure 7). This cultivar, widely grown in the Mediterranean region, is characterized by high productivity (60–65 kg per tree on average), medium-sized fruits (400–500 g), and a distinctive bright red rind covering nearly 95% of the fruit surface (Figure 6). The fruits typically ripen after mid-October, are moderately prone to cracking, but are highly susceptible to sunburn damage. Despite its sensitivity, *Hicaznar* is the predominant commercial cultivar in Türkiye, with increasing importance in both domestic production and export markets. Samples were collected and subsequent laboratory analyses were carried out using facilities of BATEM, the Department of Horticulture at Akdeniz University, and the Pathology Laboratory of Akdeniz University Faculty of Medicine. A total of 360 fruits were selected and labeled before the onset of sunburn, with five fruits sampled from each of the four cardinal directions (east, west, south, north) and from the inner canopy of every tree included in the trial. From this stage until harvest, fruit samples were collected at regular intervals and anatomical examinations were conducted. The Mediterranean Region is characterized by extremely hot and dry summers, with temperatures often reaching 35–40 °C. In the experimental orchard, temperatures ranged between 35 and 38 °C and relative humidity between 30 and 75% from June to August.

### 3.2. Sample Preparation and Fixation

Fruit peels with and without visible sunburn symptoms were excised and fixed in a formalin-acetic-alcohol (FAA) solution containing 70% ethanol (90 mL), glacial acetic acid (5 mL), and formaldehyde (5 mL), and stored at 4 °C. The fixed samples were then prepared for paraffin embedding and sectioning according to the paraffin method described by Brooks et al. [48].

In severely sunburned fruits, the hardened peel tissues made it difficult to obtain clear sections using the conventional paraffin method. Therefore, a cellulose acetate pre-treatment was applied to soften the tissues before embedding. For this purpose, samples were first immersed in distilled water to remove air bubbles, transferred into pure acetone for 2 h, and then incubated in 12% cellulose acetate solution (12 g cellulose acetate + 100 mL acetone) for 14 days. Before paraffin embedding, residual cellulose acetate was removed by briefly rinsing the samples in pure acetone.

### 3.3. Paraffin Embedding and Sectioning

Samples were dehydrated through a graded ethanol series (70%, 80%, 90%, 96%, and absolute ethanol) under vacuum, followed by clearing in a graded xylene series. Infiltration with paraffin wax was then performed at 62 °C until tissues were saturated, and samples were embedded in paraffin blocks (Supplementary Figure S1). Sections of 5–7 µm thickness were obtained using a sliding Leitz microtome (Ernst Leitz, Wetzlar, Germany) (Supplementary Figure S2). The sections were floated on a water bath (40–45 °C) to flatten the ribbons, mounted on glass slides coated with an albumin: glycerin mixture (1:1), and incubated at 60 °C for 2–3 h to ensure proper adhesion.

### 3.4. Tissue Staining and Microscopic Observation

**Figure 8:**
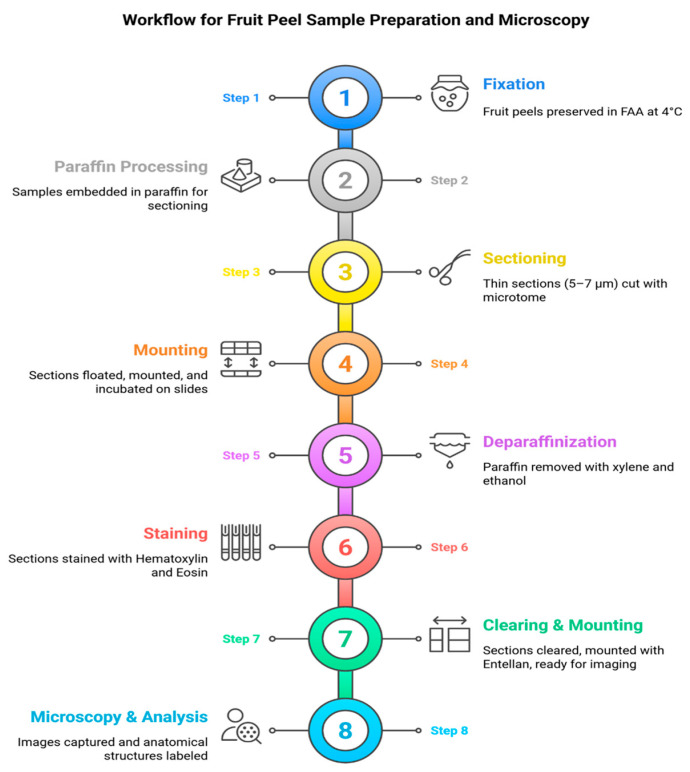
Workflow for sample preparation, fixation, staining, and microscopic observation of fruit peel sections.

Microscopic observation of thin sections requires staining. Therefore, thin sections of pomegranate peels were stained using 0.5% hematoxylin and eosin. Briefly, before staining, the thin sections were first treated with xylene for 2 min to remove paraffin. The samples were washed twice with 100% and 95% ethanol for 1 min each and then rinsed with tap water for 10 min. The thin sections were then incubated in 0.5% hematoxylin for 2 h and rinsed with tap water for 20 min. After treatment with Eosin for 2 h, the sections were washed twice with 95% ethanol and three times with 100% ethanol. Finally, the prepared sections were incubated with 100% xylene for 6 min (in 2-min intervals repeated three times) and mounted with Entellan. The stained sections were examined under a light microscope (Nikon Optiphot; Nikon Corporation, Tokyo, Japan) at various magnifications. Images of the stained sections were captured using a Nikon Coolpix 5500 digital camera (Nikon Corporation, Tokyo, Japan) attached to the microscope (Figure 8). Captured images were transferred to a computer and analyzed; observed anatomical structures were subsequently labeled.

## 4. Conclusions

Pomegranate growers in Türkiye have attempted various strategies to protect their orchards from sunburn, yet these measures have largely failed to provide adequate control. This study validates our hypothesis that the limited effectiveness of these practices is mainly due to improper timing, rather than the practices themselves. Therefore, the optimal timing for implementing protective measures against sunburn was identified and demonstrated in this study. This critical window corresponds to the stage before the fruit peel begins to develop its natural pink or red coloration. These findings highlight that anatomical alterations precede external symptoms, suggesting that preventive measures must be implemented at an early stage rather than after visible sunburn has occurred.

Beyond confirming the destructive impact of sunburn on fruit quality, this research provides novel insights into the anatomical markers of early sunburn damage in pomegranate. By identifying these structural changes, the study not only contributes to the understanding of peel anatomy under environmental stress but also offers a practical basis for developing monitoring tools and mitigation strategies in commercial orchards. The results emphasize the potential for integrating anatomical screening with preventive practices, such as shading, evaporative cooling, or protective sprays, to reduce yield and quality losses. Ultimately, this work establishes a foundation for future studies that aim to link anatomical traits with physiological resilience, thereby supporting more sustainable pomegranate cultivation under increasingly challenging climatic conditions.

In addition, these findings gain greater significance in the context of global climate change, where rising temperatures, increased exposure to solar radiation, and more frequent extreme weather events are expected to amplify the risk of sunburn damage in fruit crops. Comparative anatomical studies across different cultivars and growing environments could identify genetic or structural traits associated with greater resistance to sunburn, guiding breeding programs and cultivar selection. Expanding such research to include molecular and biochemical pathways will provide a more holistic understanding of fruit responses to abiotic stress. Therefore, the present study not only enriches the scientific knowledge on the anatomical basis of sunburn but also serves as a stepping stone toward integrated approaches that combine genetics, physiology, and orchard management to safeguard fruit quality and ensure the long-term sustainability of pomegranate production.

Taken together, these results underscore the importance of integrating anatomical insights into multidisciplinary frameworks that combine genetics, physiology, and practical orchard management. Future research should prioritize the identification of cultivar-specific anatomical traits that confer resilience, as well as the exploration of molecular pathways that regulate cellular responses to heat and light stress. Such approaches could enable the development of predictive models for sunburn risk and contribute to the design of precision agriculture tools tailored for pomegranate production. By extending the scope of research beyond individual orchards to regional and global scales, it will be possible to establish sustainable cultivation systems that minimize climate-related losses and ensure the long-term economic viability of this culturally and nutritionally valuable fruit.

From a practical perspective, the early anatomical indicators identified in this study can be directly translated into orchard management strategies. The presence of phenolic accumulation, cell-wall thickening, and initial cuticle disruption long before visible symptoms appear suggests that protective practices—such as shading nets, kaolin-based reflective sprays, evaporative cooling, and optimized irrigation scheduling should be deployed prior to the onset of external discoloration. Implementing these measures at the correct physiological stage may substantially reduce yield and quality losses in commercial orchards. The RAPD–BSA marker work previously conducted in pomegranate [47] demonstrates that stress-related traits can be supported through molecular selection. Accordingly, the anatomical indicators identified in the present study may guide future breeding efforts aimed at improving sunburn tolerance.

In addition, the anatomical traits documented here provide valuable insights for breeding programs aimed at improving sunburn tolerance. Variability in cuticle integrity, epidermal resilience, phenolic response, and lignification patterns may serve as potential selection criteria for identifying genotypes with superior resistance to heat and solar radiation. Integrating these anatomical markers with molecular and physiological screening tools could accelerate the development of new pomegranate cultivars better adapted to future climate conditions.

## Data Availability

The original contributions presented in the study are included in the article. Further inquiries can be directed to the corresponding author.

## Supplementary Materials

The following supporting information can be downloaded at: https://www.mdpi.com/article/10.3390/plants15060987/s1, Figure S1: (a) Placement of the samples into molds and paraffin embedding (b) Sealing and labeling; Figure S2: Cooled paraffin blocks removed from molds, with excess paraffin trimmed from the edges prior to storage.
