# Supplementary material for: Anatomical Changes in the Peel of Sun-Damaged Pomegranates (Punica granatum L. cv. Hicaznar)
Source: Plants (Basel). 2026 Mar 23;15(6):987. doi: 10.3390/plants15060987 (PMC13029930; doi:10.3390/plants15060987)
Supplement: Supplementary file 1 [file plants-15-00987-s001.zip › plants-3854265-supplementary.pdf]

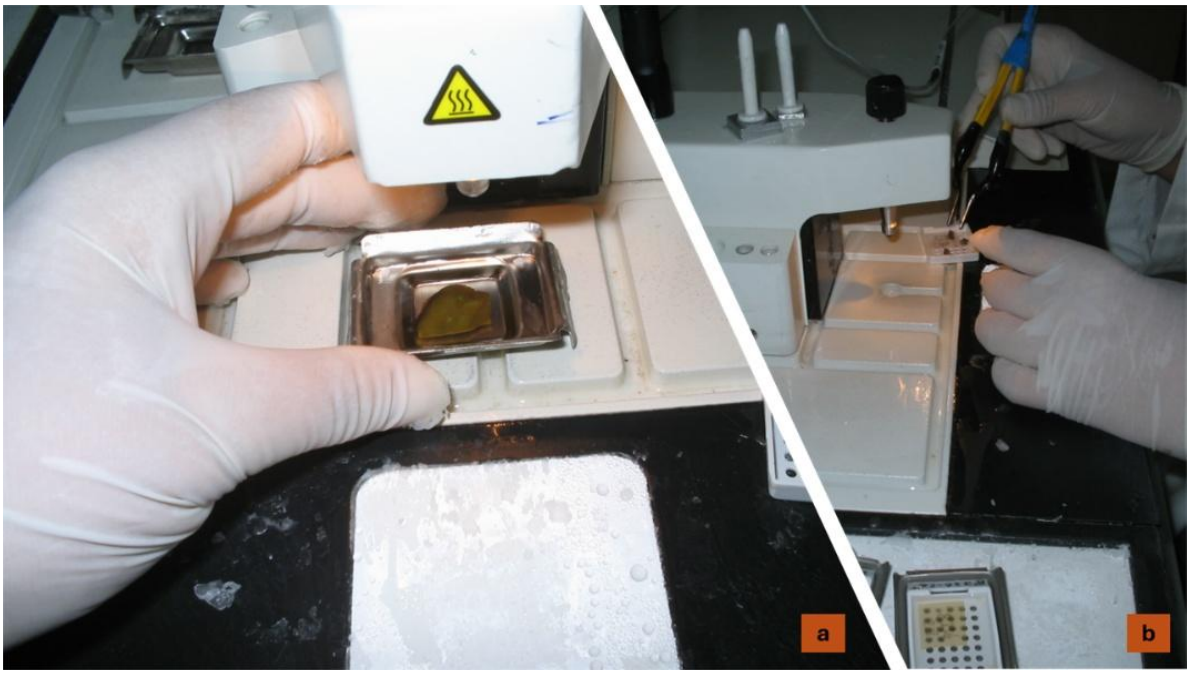


Supplementary Figure 1. Placement of the samples into molds and paraffin embedding b) Sealing and labeling

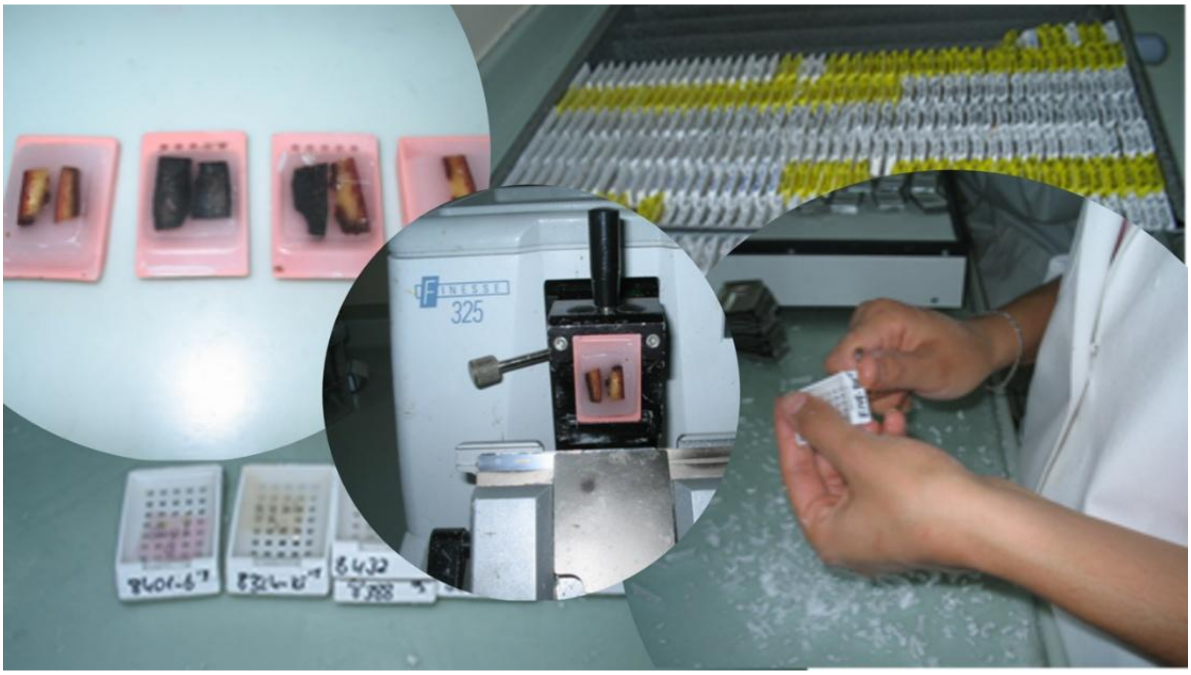


Supplementary Figure 2. Cooled paraffin blocks removed from molds, with excess paraffin trimmed from the edges prior to storage.
